# Explaining bathymetric diversity patterns in marine benthic invertebrates and demersal fishes: physiological contributions to adaptation of life at depth

**DOI:** 10.1111/brv.12061

**Published:** 2013-10-04

**Authors:** Alastair Brown, Sven Thatje

**Affiliations:** Ocean and Earth Science, University of Southampton, National Oceanography Centre SouthamptonEuropean Way, Southampton, SO14 3ZH, U.K.

**Keywords:** colonisation, deep sea, diversity, evolution, hydrostatic pressure, invertebrate, macroecology, radiation, speciation, temperature

## Abstract

Bathymetric biodiversity patterns of marine benthic invertebrates and demersal fishes have been identified in the extant fauna of the deep continental margins. Depth zonation is widespread and evident through a transition between shelf and slope fauna from the shelf break to 1000 m, and a transition between slope and abyssal fauna from 2000 to 3000 m; these transitions are characterised by high species turnover. A unimodal pattern of diversity with depth peaks between 1000 and 3000 m, despite the relatively low area represented by these depths. Zonation is thought to result from the colonisation of the deep sea by shallow-water organisms following multiple mass extinction events throughout the Phanerozoic. The effects of low temperature and high pressure act across hierarchical levels of biological organisation and appear sufficient to limit the distributions of such shallow-water species. Hydrostatic pressures of bathyal depths have consistently been identified experimentally as the maximum tolerated by shallow-water and upper bathyal benthic invertebrates at *in situ* temperatures, and adaptation appears required for passage to deeper water in both benthic invertebrates and demersal fishes. Together, this suggests that a hyperbaric and thermal physiological bottleneck at bathyal depths contributes to bathymetric zonation. The peak of the unimodal diversity–depth pattern typically occurs at these depths even though the area represented by these depths is relatively low. Although it is recognised that, over long evolutionary time scales, shallow-water diversity patterns are driven by speciation, little consideration has been given to the potential implications for species distribution patterns with depth. Molecular and morphological evidence indicates that cool bathyal waters are the primary site of adaptive radiation in the deep sea, and we hypothesise that bathymetric variation in speciation rates could drive the unimodal diversity–depth pattern over time. Thermal effects on metabolic-rate-dependent mutation and on generation times have been proposed to drive differences in speciation rates, which result in modern latitudinal biodiversity patterns over time. Clearly, this thermal mechanism alone cannot explain bathymetric patterns since temperature generally decreases with depth. We hypothesise that demonstrated physiological effects of high hydrostatic pressure and low temperature at bathyal depths, acting on shallow-water taxa invading the deep sea, may invoke a stress–evolution mechanism by increasing mutagenic activity in germ cells, by inactivating canalisation during embryonic or larval development, by releasing hidden variation or mutagenic activity, or by activating or releasing transposable elements in larvae or adults. In this scenario, increased variation at a physiological bottleneck at bathyal depths results in elevated speciation rate. Adaptation that increases tolerance to high hydrostatic pressure and low temperature allows colonisation of abyssal depths and reduces the stress–evolution response, consequently returning speciation of deeper taxa to the background rate. Over time this mechanism could contribute to the unimodal diversity–depth pattern.

## CONTENTS

- Introduction 407
- Origin of the deep-sea fauna and the colonisation of the deep sea 408
- Physiological limitation by low temperature and high hydrostatic pressure 410
- Tolerance of high hydrostatic pressure and low temperature 411
- Adaptations to high hydrostatic pressure and low temperature 413
- Bathymetric variation in evolution 415
- The stress-induced evolutionary mechanism in the deep sea 416
- Conclusions 418
- Acknowledgements 419
- References 419

## I. INTRODUCTION

**Fig. 1:**
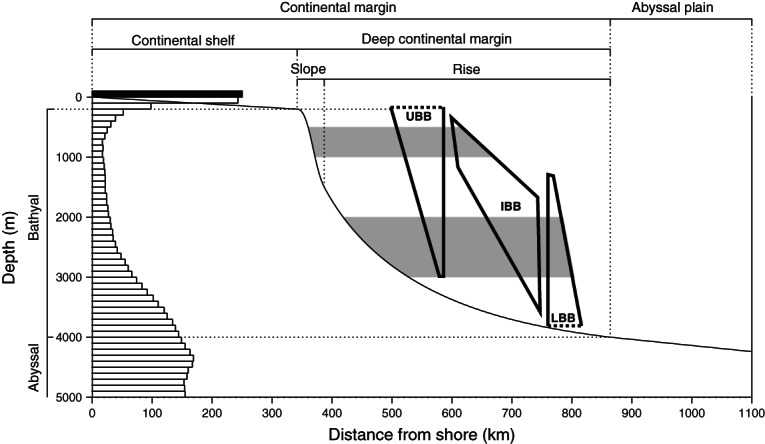
Conceptual profile of a passive aseismic continental margin (Adapted from Gage & Tyler, 1991). Horizontal bars on the left indicate percentage of total ocean surface area of each 100 m depth interval (note that this is not restricted exclusively to continental margins) estimated from figure 9.2 in Mackenzie & Lerman (2006) (black scale bar = 5%; depths greater than 5000 m not shown). In topographic terms, the continental shelf extends to the shelf break where the topographic gradient increases, characterising the continental slope. The topographic gradient reduces onto the continental rise, formed by a thick slope-derived sediment wedge, and reduces further onto the relatively flat abyssal plain. The continental margin comprises continental shelf, slope and rise; the deep continental margin comprises continental slope and rise. Ecological zones are bathyal (200–4000 m) and abyssal (4000–6000 m). Conceptual components of bathymetric patterns of diversity (Adapted from Carney, 2005; height = bathymetric range, width = species richness) are included, representing three groups of species: upper boundary biota (UBB) species extend downwards from or above the upper boundary of the deep continental margin but do not reach the lower boundary; lower boundary biota (LBB) species extend upwards from or below the lower boundary of the deep continental margin but do not reach the upper boundary; inter-boundary biota (IBB) species reach neither boundary. Shaded areas indicate depths of high species turnover consistently identified in studies of bathymetric diversity (Carney, 2005). A unimodal diversity–depth pattern typically peaks between 1000 and 3000 m despite the relatively low area represented by these depths.

Many macroevolutionary patterns display both ecological and biogeographical components. Clear bathymetric patterns have been identified in the extant biodiversity of the deep continental margins, a region covering approximately 40% of the total ocean surface area (Fig. 1) (reviewed by Merrett & Haedrich, 1997; Levin *et al.*, 2001; Stuart, Rex & Etter, 2003; Carney, 2005; Menot *et al.*, 2010; Rex & Etter, 2010). A unimodal diversity–depth pattern has been indicated by qualitative (Rex, 1981) and quantitative (Etter & Grassle, 1992) sampling studies in the western North Atlantic, the most intensively sampled region of the deep sea. Diversity appears depressed at upper bathyal depths and at abyssal depths, with a peak in diversity at intermediate depths (Rex & Etter, 2010), despite the relatively low area represented by these depths (1000–3000 m represents approximately 13% of the total ocean surface area; Fig. 1), at a level comparable to the most diverse ecosystems known (Grassle & Maciolek, 1992; Levin & Dayton, 2009). Almost all organisms are distributed between a high and a low depth limit (Pradillon & Gaill, 2007) and geometric constraints models, which stochastically place bathymetric ranges between boundaries, have yielded unimodal patterns of diversity similar to bathymetric gradients observed in the deep sea (Pineda & Caswell, 1998). However, such models cannot explain most characteristics of the parabolic bathymetric diversity pattern, i.e. curvature, or the magnitude and position of the peak (Pineda & Caswell, 1998; McClain & Etter, 2005). This unimodal diversity pattern has been attributed to varied environmental gradients, particularly in productivity and disturbance (Paterson & Lambshead, 1995; Cosson-Sarradin *et al.*, 1998; Rex *et al.*, 2005*a*), and a source–sink hypothesis has been suggested for abyssal biodiversity where abyssal populations are regulated by a balance between immigration from bathyal sources and chronic extinction arising from vulnerabilities to Allee effects (Rex *et al.*, 2005*b*, 2005). Although the first test of the source–sink hypothesis strongly suggests that source–sink dynamics contribute to the unimodal diversity pattern, it is also clear that species turnover is more important at bathyal depths (Brault *et al.*, 2012). However, the mechanisms proposed to drive the unimodal diversity–depth relationship do not consider the evolutionary history of the deep-sea fauna. Speciation rates appear to drive other biodiversity patterns (Allen & Gillooly, 2006); consequently the unimodal bathymetric diversity pattern may be influenced by discordance in the environmental pattern of evolutionary origin (Stuart & Rex, 2009). Whilst ecological processes tend to dominate over short time periods and local scales, evolutionary processes are more important over long time periods and regional or global scales (Lambshead & Boucher, 2003), and it appears that species diversity is driven largely by abiotic factors (for review see Benton, 2009). The importance of considering evolutionary processes is apparent in analysis of bathymetric zonation in the deep sea.

Although the unimodal diversity pattern seems typical of the western North Atlantic and also occurs widely in other locations, data from some geographical regions suggest that it may not be ubiquitous, being interrupted by oceanographic conditions at specific depths such as oxygen minimum zones (see Levin *et al.*, 2001; Stuart *et al.*, 2003; Menot *et al.*, 2010; Rex & Etter, 2010). The geological history of regions may also contribute to the absence of unimodal diversity patterns with depth. For example, following the Mediterranean Sea desiccation event ˜5.5 million years ago (Ma) (Krijgsman *et al.*, 1999) recolonisation by marine fauna may have been limited to shallower species by the depth of the Mediterranean sill, resulting in an impoverished Mediterranean deep-sea fauna (see Tyler, 2003) without a clear unimodal diversity pattern (Danovaro *et al.*, 2010). Regardless of the absolute global validity of the unimodal bathymetric diversity model, bathymetric patterns of species turnover and zonation in the deep sea are widespread (see Merrett & Haedrich, 1997; Carney, 2005; Menot *et al.*, 2010; Rex & Etter, 2010). Rapid depth-correlated turnover in species composition is consistently indicated at the shelf-slope transition between the shelf break and 1000 m and at the slope-abyss transition between 2000 and 3000 m. Consequently, the shelf, continental slope, and abyssal plain faunas are clearly distinct, suggesting that these transitions are biodiversity bottlenecks (Fig. 1) (Carney, 2005; Menot *et al.*, 2010). It remains uncertain which factors play a dominant role in these distributional barriers but it appears that thermal effects may contribute to bathymetric zonation patterns since temperature-related shifts in the upper transition zone have been identified, and zonal boundaries appear to become less distinct with increasing latitude (see Carney, 2005). However, bathymetric zonation persists in isothermal water columns, such as those at high latitudes or in the Mediterranean Sea (see Carney, 2005).

These diversity patterns suggest that the deep-sea margin ecosystem may offer novel contributions to ecological theory (Levin & Dayton, 2009). Here, we relate bathymetric zonation to the evolutionary history of the deep-sea fauna and to a proposed physiological mechanism of distributional limitation by high hydrostatic pressure and low temperature. We discuss the emerging pattern of hyperbaric limitation of shallow-water benthic invertebrate species and examine adaptations of deep-sea fauna to prevailing environmental conditions, to support the hypothesis that a physiological bottleneck at bathyal depths is imposed by high pressure and low temperature, and drives bathymetric zonation. We review evidence for bathymetric variation in evolutionary rate and hypothesise that a peak at bathyal depths contributes to the unimodal diversity–depth pattern over time. Finally, we hypothesise that this phenomenon may be explained by a stress–evolution mechanism in response to physiological effects of hyperbaric and thermal challenges.

## II. ORIGIN OF THE DEEP-SEA FAUNA AND THE COLONISATION OF THE DEEP SEA

The evolutionary origin and antiquity of the extant deep-sea fauna remains uncertain with the contemporary fauna appearing to comprise clades, which originated throughout the ˜541 million years (Myr) of the Phanerozoic (Jablonski *et al.*, 1983; Jablonski & Bottjer, 1991). Over these geological timescales there have been at least five (Raup & Sepkoski, 1982; see also Harnik *et al.*, 2012) relatively sudden (*c*. 1–10 Myr; Briggs, 1995) major marine extinctions, estimated to have eradicated at least half of marine species (Briggs, 2003; but see Bambach, 2006). Although some fauna appear to have survived these events (see e.g. Thuy *et al.*, 2012), in the most severe case approximately half of all marine families (Sepkoski, 1986) and more than 95% of marine species (Raup, 1979; Benton & Twitchett, 2003) disappeared. Consequently, it has been suggested that climate-driven dysoxic extinction events in the deep sea and subsequent recolonisations have occurred on multiple occasions (Wignall & Twitchett, 1996; Horne, 1999; Wilson, 1999; Rogers, 2000; Kiehl & Shields, 2005; Wignall, Newton & Little, 2005). General onshore–offshore patterns of evolution have been reported from extensive analyses of the fossil record of shelf communities of the Phanerozoic: higher taxonomic level innovation occurred predominantly in nearshore settings before expanding into offshore environments, while rates of genera-level evolution appear to be diversity dependent, shaped by clade-specific bathymetric gradients (Jablonski, 2005, and references cited therein). For example, scleractinian corals appear to have originated ˜237 Ma in shallow water (see Jablonski & Bottjer, 1991) before invading the deep sea (Kitahara *et al.*, 2010) perhaps on several occasions during the last 65.5 Myr (Os'kina, Keller & Nikolaev, 2010). Similarly, molluscs appear to have made invasions of the deep sea from multiple shallow-water regions, although no families or higher groups of mollusc appear to have originated in the deep sea (Clarke, 1962; Allen, 1978). Deep-sea fishes, too, appear to have originated in shallow water before colonising the deep sea during the last ˜70 Myr (see Merrett & Haedrich, 1997, and references cited therein). For example, the fossil record of gadoid and macrouroid fishes suggests origination in a shallow continental shelf environment, but with adaptation to deep-water settings early in their evolution prior to radiation (Merrett & Haedrich, 1997; Kriwet & Hecht, 2008, and references therein).

Phylogenetic analyses have supported the relatedness of extant deep-sea and shallow-water species, predominantly consistent with diversification and invasion of deep-sea environments from shallow water, albeit over differing timescales. Bresiliid shrimp from deep-sea vents and seeps are reported to have radiated less than 20 Ma and from shallow ancestry (Shank *et al.*, 1999; Tokuda *et al.*, 2006). Similarly, vesicomyid clams appear to have invaded the deep sea from coastal habitats between 22 and 44 Ma, occupying cold seep habitats before colonising hydrothermal vent environments (Little & Vrijenhoek, 2003; Decker *et al.*, 2012). Indeed, other vent and seep fauna also originated relatively recently in geological terms, within the last 100 Myr (see Little & Vrijenhoek, 2003).

Pagodulid snails appear to have radiated even more recently, from a shallow-water Antarctic lineage, and colonised the deep sea approximately 3 Ma (Barco *et al.*, 2012). However, colonisation of the deep sea by shallow-water Antarctic fauna is not exclusively recent. The notothenioid fish species flock also appears to have radiated in the last ˜21 Myr (Bargelloni *et al.*, 2000), evolving in Antarctic shallow water before invading the deep sea (see Clarke & Johnston, 1996, and references therein). Molecular phylogenetic evidence indicates that a deep-sea octopus lineage invaded from shallow-water Antarctic origin, diverging around 33 Ma and subsequently radiating 15 Ma (Strugnell *et al.*, 2008). Similarly, nudipleuran evolution is proposed to have taken place around the cooling of Antarctica about 40 or 30 Ma prior to invasion of temperate and tropical seas along northward flowing currents of Antarctic origin (see Göbbeler & Klussmann-Kolb, 2010). Fossil evidence also suggests submergence of shallow-water Antarctic bivalves, gastropods, asteroids, crinoids and decapods into the deep sea during this period (Zinsmeister & Feldmann, 1984). Although the timing remains unclear, molecular evidence indicates that palinurid spiny lobsters originated around Antarctica, invading deep-sea habitats from shallower rocky reefs and then radiating (Tsang *et al.*, 2009).

Palaeontological and molecular data indicate that echinoids have made migrations to the deep sea over multiple timescales; generalist omnivores migrated to the deep sea in low numbers over the last 200 Myr in contrast to the majority of specialist detritivore clades, which made independent off-shelf migrations between approximately 75 and 55 Ma (Smith & Stockley, 2005). Similarly, deep-sea asellote isopods originate from at least four major and independent migrations from shallow water: however, these isopods are proposed to have invaded the deep sea prior to the dysoxic events during the end-Permian extinction event *ca*. 250 Ma (see Raupach *et al.*, 2009; Lins *et al.*, 2012). Torquaratorid acorn worms also appear to have colonised the deep sea prior to ˜250 Ma, invading from shallow water before demonstrating an extensive radiation *in situ* (Osborn *et al.*, 2012). Bivalve molluscs were well represented in the Ordovician (485–443 Ma) but also show evidence of more recent radiations (Allen, 1978), e.g. deep-sea bathymodiolinid mussels found at hydrothermal vents and cold seeps represent a recent evolutionary radiation from modern shallow-water mytilid taxa as organic fall specialists (Distel *et al.*, 2000; Lorion *et al.*, 2010), estimated to have occurred 21 Ma (Miyazaki *et al.*, 2010). The deep-water lithodid crabs also appear to have originated recently, with at least three radiations from North Pacific shallow-water ancestors since the evolution of the lithodids (Hall & Thatje, 2009) between 13 and 25 Ma (Cunningham, Blackstone & Buss, 1992; but see McLaughlin, Lemaitre & Sorhannus, 2007). The potential for depth-range extension and colonisation of the deep sea by shallow-water species persists; there is evidence that the echinoid sea urchin *Echinus acutus* is extending its bathymetric range, indicating that migrations to the deep sea are still occurring (Tyler & Young, 1998; Minin, 2012).

Reemergence from the deep sea has also been reported, e.g. for lithodid crabs (Hall & Thatje, 2009) and possibly for cylindroleberidid ostracods (Syme & Oakley, 2012; see their discussion for contrasting conclusions from different analytical methods). Further, some taxa originated in the deep sea and ascended to shallow water. Following origination ˜62 Ma (Bernecker & Weidlich, 1990), molecular phylogeny indicates stylasterid corals diversified extensively in the deep sea before making three distinct invasions of the shallow-water tropics and a single invasion of temperate shallow water (Lindner, Cairns & Cunningham, 2008). Similarly, chrysogorgiid soft corals and pennatulid sea pens appear to have originated in the deep sea before radiating globally and into shallow water (Dolan, 2008; Pante *et al.*, 2012). Molecular phylogeny indicates that freshwater eels also originated in the deep ocean following invasion from shallow water, reflecting their evolutionary origin in their catadromous life cycle (Inoue *et al.*, 2010). However, examples of origination of higher taxonomic levels in the deep sea are relatively few, and the extant deep-sea fauna is considered to result predominantly from both ancient and more recent radiations of shallow-water lineages into deep water (Horne, 1999; Wilson, 1999).

Since shallow-water fauna are adapted to relatively warm conditions currently dominating the upper oceans except at high latitudes, the low temperatures prevalent in the deep sea are considered to limit invasion by such fauna (deep-sea temperature is typically between 4 and −1°C; Gage & Tyler, 1991). Consequently, it is believed that the colonisation of the deep sea may have been limited to periods and regions with an isothermal water column (Tyler, Young & Clarke, 2000). Warm water columns are currently restricted to isolated seas, e.g. water temperature is 21.5°C at 2 km depth in the Red Sea and is 13°C at 4 km depth in the Mediterranean Sea (Gage & Tyler, 1991), but were widespread in some earlier geological periods. For example, the vertically homogenous warm ocean of the late Mesozoic and early Cenozoic (with deep-sea bottom temperatures up to 16°C; Lear, Elderfield & Wilson, 2000; Zachos *et al.*, 2002; Cramer *et al.*, 2011) could have permitted invasion of deep water, later requiring adaptation to cold temperatures as the oceans gradually evolved to the current psycrospheric state (Young, Tyler & Fenaux, 1997; Thatje, Hillenbrand & Larter, 2005). Invasion of the deep sea by the majority of specialist detritivore echinoids occurred during this period (Smith & Stockley, 2005). At other geological times near-isothermal cold water columns in regions of deep-water formation at high latitudes have presented an opportunity for deep-sea invasion. Molecular phylogeny has indicated Antarctic shallow water as the origin of both deep-sea asellote isopods (>250 Ma; Raupach *et al.*, 2009) and deep-sea octopus (˜33 Ma; Strugnell *et al.*, 2008) during periods with low-temperature deep-water formation at high latitudes (Horne, 1999), prior to deep-sea radiation.

In both warm and cold isothermal water columns the major limiting factor for range extension into the deep sea is predicted to be tolerance of high hydrostatic pressure (Young *et al.*, 1997; Thatje *et al.*, 2005). Phylogenetic and physiological studies have certainly emphasised thermal and hyperbaric bottlenecks in an evolutionary context, with passage to deeper water requiring adaptation to low temperatures and high hydrostatic pressures (Macdonald, 1972; Menzies & George, 1972; Macdonald & Teal, 1975; George, 1979; Hall & Thatje, 2009; Mestre, Thatje & Tyler, 2009; Thatje, Casburn & Calcagno, 2010; Brown & Thatje, 2011; Oliphant *et al.*, 2011; Smith & Thatje, 2012). Evidence of critical biological effects of hydrostatic pressure and temperature could support the imposition of limits on bathymetric distribution by these factors.

## III. PHYSIOLOGICAL LIMITATION BY LOW TEMPERATURE AND HIGH HYDROSTATIC PRESSURE

Thermal tolerance is proposed to relate directly to the physiological ability of an organism to avoid the transition from aerobic to anaerobic metabolism, with a systemic to molecular hierarchy of limitation (see Pörtner, 2001, 2002). Under environmental conditions beyond optimum, the homeostatic effort required to maintain internal conditions within physiological tolerance boundaries increases. Low temperatures have been shown to interrupt protein structure for numerous proteins (for review see Privalov, 1990; Kunugi & Tanaka, 2002; Marqués *et al.*, 2003). Molecular and physiological studies of cold stress suggest that this can result in elevated protein chaperoning in response to cold denaturation (Place & Hofmann, 2005; Schmid *et al.*, 2009). These protein chaperones counteract the stabilisation of the secondary structures of RNA and DNA and the consequent reduction in the efficiency of translation, transcription, and DNA replication (Phadtare, Alsina & Inouye, 1999), and may be required for ribosome assembly at suboptimal temperatures (Gualerzi, Giuliodori & Pon, 2003). Low temperature also decreases the fluidity of biological membranes, significantly reducing membrane function (Hazel, 1995). Mitochondrial activity increases to facilitate the increased homeostatic effort, however increased mitochondrial oxygen demand is not directly matched by increased respiratory capacity delivered through ventilation and circulation (e.g. Frederich & Pörtner, 2000). Subsequently, a transition from aerobic to anaerobic mitochondrial respiration occurs at the critical threshold where mitochondrial oxygen demand exceeds the respiratory capacity of the animal; survival under such conditions is time limited. The effects of oxygen limitation on the cardiac muscle are amplified as mitochondrial oxygen demand increases ultimately forming a positive feedback loop (Somero, 2005). Mitochondrial densities and their functional properties appear to be critical in defining thermal tolerance windows, e.g. at low temperatures the aerobic capacity of mitochondria may become limiting for ventilation and circulation. Adjustments in mitochondrial densities and functional properties can shift temperature envelopes tolerated by organisms (Sommer & Pörtner, 2002). However, integrated molecular modifications in lipid saturation, kinetic properties of metabolic enzymes, contractile proteins, and transmembrane transporters are also essential for maintaining higher functions (Pörtner, 2002). Outside the optimal range basic metabolic processes can be maintained before the critical threshold, but non-essential processes such as growth, reproduction, feeding, and voluntary movement are reduced (Cossins & Bowler, 1987; Peck, 1998; Peck, Webb & Bailey, 2004; Pörtner, 2004; Young, Peck & Matheson, 2006; Peck *et al.*, 2007, 2008). At a species level diminished aerobic scope induced by environmental factors may have significant impacts; reductions in growth and reproductive output will affect the survival of species (Pörtner, 2002). Complex animals rely on ventilation and circulatory systems to supply their cells with oxygen, and consistent with this oxygen-limitation hypothesis inter- and intraspecific analyses have indicated that smaller individuals survive to higher temperatures than larger ones in marine species and that more active species survive higher elevated temperatures (Peck, Pörtner & Hardewig, 2002; Pörtner, 2002; Peck *et al.*, 2004, 2007, 2009 Pörtner, Peck & Hirse, 2006; Pörtner, Peck & Somero, 2007). This may explain the apparent preferential survival of small species during extinction events (Cardillo, 2003).

There are significant physical effects of hydrostatic pressure on proteins and lipoprotein membranes (reviewed by Pradillon, 2012). Relatively moderate pressure increase may induce protein subunit dissociation, and consequently denaturing of enzymes (for review see Gross & Jaenicke, 1994; Mozhaev *et al.*, 1996; Boonyaratanakornkit, Park & Clark, 2002; Winter & Dzwolak, 2005). For example, macromolecular protein assemblages such as cytoskeleton tubulin and actin are dissociated by pressure in the range of a few tens of MPa in shallow-water organisms, affecting basic cell morphology and organisation (Kennedy & Zimmerman, 1970; Salmon, 1975*a*,*b*; Begg, Salmon & Hyatt, 1983; Swezey & Somero, 1985; Bourns *et al.*, 1988). Synthesis of proteins is also susceptible to elevated pressure (Gross & Jaenicke, 1994). Lipid bilayers of biological membranes appear one of the most pressure-sensitive molecular assemblages (Wann & Macdonald, 1980; DeLong & Yayanos, 1985; Somero, 1992; Macdonald, 1997; Winter & Dzwolak, 2005); pressure increase orders structures and reduces flexibility in lipids, nucleic acids and carbohydrates (Behan *et al.*, 1992; Balny, Masson & Heremans, 2002). An increase in pressure of 100 MPa is equivalent to a decrease in temperature of approximately 13–21°C depending on membrane composition (Somero, 1992); a temperature increase of 2.8°C has been reported to reverse the reduction in membrane fluidity imposed by a hydrostatic pressure of 10 MPa (De Smedt *et al.*, 1979). The effects of reduced membrane functionality on action potential transmission in nervous cells (Wann & Macdonald, 1980; Siebenaller & Garrett, 2002) are clearly visible as a high-pressure neurological syndrome in organisms exposed to pressures radically different from those within their natural distribution; signs are motor coordination impairment, spasm and even paralysis (Menzies & George, 1972; Macdonald & Teal, 1975; Wilcock, Wann & Macdonald, 1978; Yayanos, 1981; Avrova, 1984; Heinemann *et al.*, 1987; Treude *et al.*, 2002; Oliphant *et al.*, 2011). The interference can affect cardiac function (Mickel & Childress, 1982*b*; Airriess & Childress, 1994), with clear implications for aerobic scope. Observed respiratory and cardiac responses to pressure change appear to support the application of the oxygen-limitation hypothesis to hydrostatic pressure tolerance (George, 1979; Mickel & Childress, 1982*a*; Robinson, Thatje & Osseforth, 2009; Brown & Thatje, 2011; Thatje & Robinson, 2011), with further consistent indications that voluntary movement and feeding are affected by hyperbaric conditions beyond optimum (Thatje *et al.*, 2010; Thatje & Robinson, 2011). Aerobic scope certainly appears the crucial factor setting tolerance limits (Peck *et al.*, 2002, 2004, 2009; Pörtner, 2002; Pörtner *et al.*, 2006, 2007; Brown & Thatje, 2011; Thatje & Robinson, 2011).

Cellular responses to thermal and hyperbaric environmental challenges can contribute directly to biogeographic limitation (e.g. see Tomanek, 2010). The effects of increases in pressure and temperature on proteins and lipid bilayers of biomembranes are largely antagonistic within ecologically relevant ranges (Balny, Mozhaev & Lange, 1997; Winter & Dzwolak, 2005), suggesting that low temperatures and high pressures may act together in limiting bathymetric distribution of species (e.g. Brown & Thatje, 2011). Cells of atmospheric-pressure-adapted organisms respond to pressure changes by altering synthetic capacity (Parkkinen *et al.*, 1993; Lammi *et al.*, 1994; Smith *et al.*, 1996). Analysis of transcription in articular cartilage cells indicates up-regulation of mRNA of several genes mediating growth arrest (Sironen *et al.*, 2002), and exposure to high pressure causes cellular growth arrest (Abe & Horikoshi, 2000; Koyama *et al.*, 2005) and decreased levels of mRNA of genes involved in cell-cycle progression (Fernandes *et al.*, 2004). Reduced levels have also been reported for genes involved in protein synthesis (Fernandes *et al.*, 2004; Elo *et al.*, 2005). However, cells exposed to continuous high hydrostatic pressure regimes have also responded by up-regulating several heat shock proteins (Kaarniranta *et al.*, 1998, 2000; Elo *et al.*, 2000, 2005). This response protects proteins from acute and chronic stress by stabilising and refolding protein-folding intermediates or by facilitating protein degradation (Morimoto *et al.*, 1997), and has recently been reported in atmospheric-pressure-adapted shrimp exposed to high hydrostatic pressure and without onset of systemic failure (Cottin *et al.*, 2012). Similarly, cold stress can result in elevated protein chaperoning in response to cold denaturation (Place & Hofmann, 2005; Schmid *et al.*, 2009). Expression of cold shock proteins can also be induced in organisms exposed to increased hydrostatic pressure (e.g. Welch *et al.*, 1993; Wemekamp-Kamphuis *et al.*, 2002). Considering the analogous effects of high hydrostatic pressure and low temperature, such responses may be critical to colonising the deep sea; the cold shock response of the microorganism *Listeria monocytogenes* after exposure to 10°C for 4 h following culture at 37°C, has been reported to result in a 100-fold increase in survival of exposure to 300 MPa for 20 min (Wemekamp-Kamphuis *et al.*, 2002).

Evidently, hydrostatic pressure and temperature both have significant biological effects perturbing every level of biological organisation sufficiently to limit biogeographic range (Table 1). It appears likely that an organism's capacity for survival at any given depth is determined by the sum of hydrostatic pressure and temperature interactions (Sébert, 2002) in advance of other ecological considerations. Experimental evidence assessing hyperbaric limitation across a range of shallow-water benthic invertebrate taxa at *in situ* temperatures at bathyal depths could support the contribution of hydrostatic pressure to bathymetric zonation.

**Table 1:** Proposed timescales and known physiological effects of high hydrostatic pressure and low temperature, and responses across hierarchical levels of organisation (see Sections III and VII)

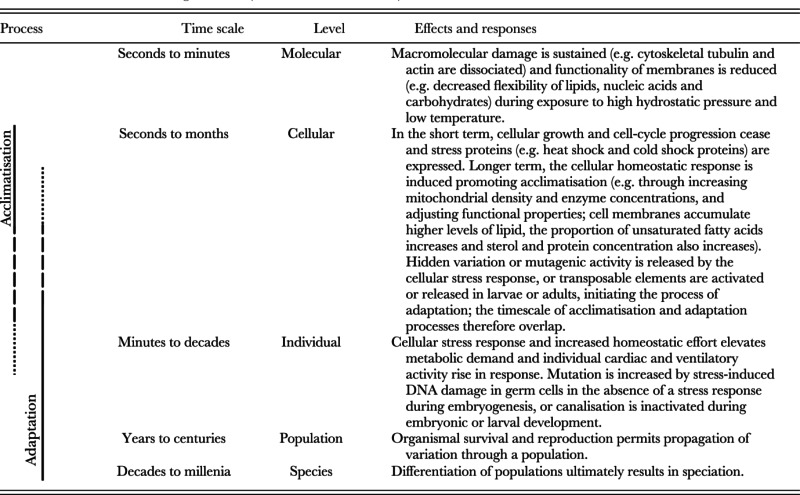

## IV. TOLERANCE OF HIGH HYDROSTATIC PRESSURE AND LOW TEMPERATURE

Recently, attempts to determine potential for invasion of the deep sea have focused on mollusc and echinoderm propagule tolerance of hydrostatic pressure and low temperature in shallow-water species, with and without close phylogenetic links to deep-sea species, in order to test the validity of theories of deep-sea colonisation (Young, Tyler & Emson, 1995; Young, Tyler & Gage, 1996; Young *et al.*, 1997; Tyler & Young, 1998; Tyler *et al.*, 2000; Benitez Villalobos, Tyler & Young, 2006; Aquino-Souza, Hawkins & Tyler, 2008; Mestre *et al.*, 2009; Smith & Thatje, 2012; Mestre, Brown & Thatje, 2013). In all cases these studies have indicated impressive pressure tolerances considerably beyond those experienced in the known adult distributions of the study species. However, although juveniles of several echinoderm species are known to settle outside of the adult bathymetric range these animals do not normally survive; growth in these individuals is reported to be slower suggesting that temperature and/or pressure may be important contributing factors (Gage & Tyler 1981*a*,*b*; Sumida *et al.*, 2000; Sumida, Tyler & Billett, 2001; Howell, Billett & Tyler, 2002). These studies also suggest that hydrostatic pressure tolerance is influenced by species' thermal adaptive history. Experimental evidence assessing tolerance to hydrostatic pressure indicates that organismal tolerance to the effects of high hydrostatic pressure can vary through ontogeny (George, 1984; Young *et al.*, 1997; Tyler & Young, 1998; Tyler *et al.*, 2000; Aquino-Souza, 2006; Benitez Villalobos *et al.*, 2006; Yoshiki *et al.*, 2006, 2008, 2011; Aquino-Souza *et al.*, 2008; Smith & Thatje, 2012; Mestre *et al.*, 2013). It appears that tolerance increases following early cleavages and subsequently decreases through further life-history stages (e.g. Tyler *et al.*, 2000). A mechanistic model has been proposed to explain this pattern (see Mestre *et al.*, 2013). The absence of a stress response during embryogenesis, and associated inability to counteract the effects of high pressure, may cause early intolerance of pressure. The increasing ability of larvae to express such a stress response may yield the subsequent increases in tolerance. Increasing difficulty in maintaining oxygen supply with greater organismal complexity and size, in the absence of adaptations to high hydrostatic pressure, may result in the following decreases in tolerance. The only investigation of hyperbaric pressure tolerance through embryonic, larval, juvenile and adult life-history stages suggests that such differential tolerances can drive ontogenetic bathymetric migrations in the Antarctic krill *Euphausia superba* (George, 1984). Whilst knowledge of larval tolerance to hydrostatic pressure and/or temperature may be critical to understanding dispersal pathways and may contribute, for example, to theories regarding hydrothermal vent and cold seep colonisation (Tyler & Dixon, 2000; Brooke & Young, 2009; Arellano & Young, 2011), it is clear that studies involving adult organisms are also essential to understanding bathymetric patterns of biodiversity and evolution. Indeed, adult-specific genes have experienced greater positive selection than those expressed in larvae in the urchin *Allocentrotus fragilis* during adaptation to the deep-sea environment (Oliver *et al.*, 2010). Recent studies have highlighted the importance of holistic investigations evaluating the physiological effects of pressure in a variety of routine behaviours. For example, the metabolic requirements of feeding in the shallow-water crab *Maja brachydactyla* appear to be greater under hyperbaric conditions, potentially critical in restricting bathymetric distributions (Thatje & Robinson, 2011).

Thorough investigation of both temperature and hydrostatic pressure tolerances of adult specimens of shallow-water species are few and focus on crustaceans, but have also demonstrated tolerance of pressures outside known natural distributions (Naroska, 1968; Menzies & George, 1972; Macdonald & Teal, 1975; George, 1979; Thatje *et al.*, 2010; Oliphant *et al.*, 2011; Thatje & Robinson, 2011; Cottin *et al.*, 2012). For example, the shallow-water shrimp *Palaemonetes varians* tolerates pressures equivalent to 1000 m depth for at least a month and retains the ability to feed and successfully moult at this pressure, despite naturally inhabiting depths of less than 10 m (Cottin *et al.*, 2012). Interaction of temperature and pressure effects has also been identified in behavioural and molecular responses of this species, with lower temperature reducing critical pressure tolerance and stimulating a significant molecular stress response at pressure equivalent to 1000 m depth (Oliphant *et al.*, 2011; Cottin *et al.*, 2012). Only a single organism-level study has extensively examined the interaction of hydrostatic pressure and temperature effects on a deep-sea species. Respiratory measurements suggest that the 2000 m lower bathymetric limit of the bathyal lysianassoid amphipod *Stephonyx biscayensis* is determined by the combination of low-temperature and high-pressure effects (Brown & Thatje, 2011). Beyond this maximum depth limit oxygen consumption is significantly reduced, indicating that oxygen supply is functionally limited and suggesting that this restricts the bathymetric range of this species. Seasonal acclimatisation to low temperature appears to increase tolerance to hydrostatic pressure (Naroska, 1968) implying that the requirements for thermal and hyperbaric acclimatisation may be congruent. Similar variation in hydrostatic pressure tolerance has been reported for latitudinally distinct populations of a single species, although genetic variation may contribute to this pattern (Aquino-Souza, 2006). Surprisingly, however, limited research has so far indicated that critical pressure tolerance is unaffected by hydrostatic pressure acclimation (Mickel & Childress, 1982*a*; Brown & Thatje, 2011).

**Fig. 2:**
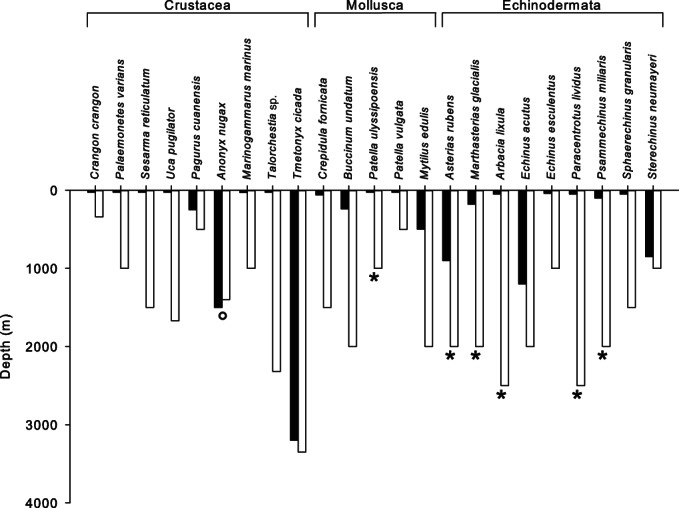
Experimentally determined hydrostatic pressure tolerances (white bars) and reported adult bathymetric distributions of shallow-water benthic invertebrate species (black bars). Tolerances presented are for the most developmentally advanced stage examined at ecologically relevant temperatures and are determined by a variety of measures (see Table 2 for details). Studies using coarse measures or temperatures not ecologically relevant are excluded. Asterisks indicate tolerance of the highest hydrostatic pressure assessed. °, Note that the slight discrepancy in hydrostatic pressure tolerance and adult bathymetric distribution of *Anonyx nugax* is likely to result from the resolution of pressure treatments used to assess hydrostatic pressure tolerance. Maximum tolerance is consistently identified at bathyal pressures, indicating that temperature and pressure equating to these depths may impose a physiological bottleneck at bathyal depths on shallow-water fauna colonising the deep sea following mass extinctions. This coincides with high bathymetric turnover of species, suggesting that the hyperbaric and thermal physiological bottleneck contributes to bathymetric zonation.

Existing studies of hyperbaric pressure tolerance of shallow-water benthic invertebrates consistently indicate limitation at bathyal depths (Fig. 2; Table 2), coinciding with regions of high species turnover. This pattern is constrained by studies on larval molluscs and echinoderms and adult crustaceans, demanding caution in adopting this as a model for other taxa. However, this model suggests that a physiological bottleneck for colonising shallow-water organisms may contribute to establishing bathymetric zonation. Since the onset of hyperbaric effects can occur at lower pressure at low temperature (Thatje *et al.*, 2010; Brown & Thatje, 2011; Oliphant *et al.*, 2011) this may explain decreasingly distinct and blurring bathymetric zonal boundaries with increasing latitude, despite the persistence of such boundaries even in the Antarctic (e.g. Kaiser *et al.*, 2011). It is recognised that challenges across biological scales can drive evolution over variable timescales (e.g. Peck, 2011), and adaptive traits appear to define the limits of species distributions and to affect demographic dynamics significantly (Carnicer *et al.*, 2012). It therefore appears likely that invasion of the deep sea by shallow-water taxa must promote adaptation to the effects of the high-hydrostatic-pressure and low-temperature environmental conditions. Evidence of such adaptation would provide further support for the role of these environmental factors in establishing bathymetric zonation.

**Table 2:** Shallow-water benthic invertebrate species assessed for elevated hydrostatic pressure tolerance, indicating the most developmentally advanced ontogenetic stage assessed, pressure treatment (pressures assessed, rate of pressurisation and duration of exposure), tolerance measure (B, behaviour; R, respiration; D, development), and maximum pressure treatment tolerated (*P*_tol_) at an ecologically relevant temperature (*T*) determined from bathymetric profiles presented by Locarini *et al.* (2010). Studies using coarse measures or temperatures not ecologically relevant are excluded

Taxon	Stage	Pressure treatment (MPa)	Measure	*P*_tol_ (MPa)	*T* (°C)	Reference
Crustacea						
*Crangon crangon*	Adult	0.1–20 stepwise (1 6 m^−1^)	B	3.4	8	Wilcock *et al.* (1978)
*Palaemonetes varians*	Adult	0.1–30 stepwise (1 5 m^−1^)	B	10	5	Oliphant *et al.* (2011)
*Sesarma reticulatum*	Adult	0.1–207 ramped (0.98 s^−1^)	B	15	10	Menzies & George (1972)
*Uca pugilator*	Adult	0.1–207 ramped (0.98 s^−1^)	B	16.7	10	Menzies & George (1972)
*Pagurus cuanensis*	Adult	0.1, 2, 5, 10 acute; 1 h	R	5	10	Thatje *et al.* (2010)
*Anonyx nugax*	Adult	0.1, 6, 14, 20, 25 ramped (0.4 m^−1^), 4 h	B, R	14	−1	George (1979)
*Marinogammarus marinus*	Adult	0.1–40 stepwise (5 5 m^−1^)	B	10	3	Macdonald (1972)
*Talorchestia* sp.	Adult	0.1–207 ramped (0.98 s^−1^)	B	23.2	10	Menzies & George (1972)
*Tmetonyx cicada*	Adult	0.1–50 stepwise (5 5 m^−1^)	B	33.5	6	Macdonald & Gilchrist (1978)
Mollusca						
*Crepidula fornicata*	Larva	0.1, 5, 10, 15, 20, 25, 30, 35, 40	B	15	10	Mestre *et al.* (2013)
*Buccinum undatum*	Juvenile	0.1, 10, 20, 30, 40 acute; 4 h	R	20	6	Smith & Thatje (2012)
*Patella ulyssipoensis*	Larva	0.1, 5, 10 acute; 24 h	B	10	10	Aquino-Souza (2006)
*Patella vulgata*	Larva	0.1, 5, 10 acute; 24 h	B	5	10	Aquino-Souza (2006)
*Mytilus edulis*	Embryo	0.1, 10, 20, 30, 40, 50 acute; 24 h	D	20	10	Mestre *et al.* (2009)
Echinodermata						
*Asterias rubens*	Larva	0.1, 5, 10, 15, 20 acute; 24 h	B	20	5	Benitez Villalobos *et al.* (2006)
*Marthasterias glacialis*	Larva	0.1, 5, 10, 15, 20 acute; 24 h	B	20	5	Benitez Villalobos *et al.* (2006)
*Arbacia lixula*	Larva	0.1, 5, 15, 25 acute; 20 h	B	25	10	Young *et al.* (1997)
*Echinus acutus*	Larva	0.1, 10, 20, 25 acute; 24 h	B	20	4	Tyler & Young (1998)
*Echinus esculentus*	Larva	0.1, 10, 20, 25 acute; 24 h	B	10	4	Tyler & Young (1998)
*Paracentrotus lividus*	Larva	0.1, 5, 15, 25 acute; 20 h	B	25	10	Young *et al.* (1997)
*Psammechinus miliaris*	Larva	0.1, 5, 10, 15, 20 acute; 24 h	B	20	5	Aquino-Souza *et al.* (2008)
*Sphaerechinus granularis*	Larva	0.1, 5, 15, 25 acute; 20 h	B	15	10	Young *et al.* (1997)
*Sterechinus neumayeri*	Larva	0.1, 5, 10, 15, 20, 25 acute; 24 h	B	10	0.9	Tyler *et al.* (2000)

## V. ADAPTATIONS TO HIGH HYDROSTATIC PRESSURE AND LOW TEMPERATURE

The oxygen requirements of cold and deep-living species do not appear to be elevated (Childress *et al.*, 1990; Peck & Conway, 2000; Drazen & Seibel, 2007; Seibel & Drazen, 2007) suggesting they are functionally adapted to high hydrostatic pressure and low temperature (Childress, 1995). Adaptation to environmental conditions can be demonstrated through comparison of natural populations of related taxa (see Franks & Hoffman, 2012). Increased mitochondrial concentration, enzyme concentration, adoption of enzymes with greater efficacy at low temperatures, and inclusion of modulator compounds that facilitate enzyme reactions, are all strategies identified in successful adaptation to cold habitats (Hazel, 1995; Clarke, 1998). Enzyme adaptation and modulation has increased in importance in updated cold-adaptation models (Clarke, 1998; Hochachka & Somero, 2002; Somero, 2003). *In vitro* evidence indicates that critical enzyme functionality can be maintained under different pressure and temperature regimes by changes of relatively few amino acids at critical positions in a protein chain, or by the inclusion of stabilizing compounds in the intracellular matrix (Carney, 2005). Indeed, positive selection of genes involved in metabolism is reported in the deep-sea urchin *Allocentrotus fragilis*, in contrast to selection in the shallow-water urchin *Stronglyocentrotus purpuratus* (Oliver *et al.*, 2010). Three categories of dehydrogenases have shown functional depth adaptation (Somero, 1998) and the importance of enzyme-stabilising compounds has been confirmed for the osmolyte trimethylamine N-oxide, which counteracts the effects of pressure by increasing cell volume (Yancey & Siebenaller, 1999; Samerotte *et al.*, 2007). Low molecular weight compounds mediating pressure effects have been reported from a variety of deep-sea fish, invertebrates and microbes (Kelly & Yancey, 1999; Yancey & Siebenaller, 1999; Yin *et al.*, 1999, 2000; Yancey *et al.*, 2000, 2004; Yin & Yancey, 2000; Fiess *et al.*, 2001, 2002; Martin, Bartlett & Roberts, 2002; Siebenaller & Garrett, 2002; Yancey, Blake & Conley, 2002; Yancey, 2005). Similarly, functional depth adaptation has been identified in cytoskeletal actin and tubulin filaments (Morita, 2003, 2004; Koyama *et al.*, 2005), with associated increase in thermal stability (Swezey & Somero, 1982). Positive selection of genes involved in skeletal development in the deep-sea urchin *Allocentrotus fragilis* contrasts with selection in the shallow-water urchin *Stronglyocentrotus purpuratus* and may reflect adaptation to environmental effects of the deep-sea (Oliver *et al.*, 2010).

The reduced fluidity of bio-membranes under increased hydrostatic pressure and decreased temperature necessitates homeoviscous adaptations in membrane structure and composition (Hazel & Williams, 1990; Balny *et al.*, 2002). Accumulation of higher levels of lipid and an increased proportion of unsaturated fatty acids have also been observed, counteracting pressure- and temperature-induced decrease in membrane fluidity (White & Somero, 1982; Avrova, 1984; Cossins & Macdonald, 1984; Macdonald, 1984; DeLong & Yayanos, 1985, 1986; Phleger & Laub, 1989; Hazel, 1995; Sébert, Theron & Vettier, 2004). Membrane fluidity may also be maintained by adjusting concentration of sterols or proteins (Winter & Dzwolak, 2005). Protein adaptations similar to those proposed in Somero's (2003) descriptive model have been identified in the protein component of membranes, where shifts in pressure induce changes in transmembrane signalling (Siebenaller & Garrett, 2002; Campanaro, Treu & Valle, 2008). Positive selection of genes involved in endo- and exocytosis in the deep-sea urchin *Allocentrotus fragilis* is in contrast to selection in the shallow-water urchin *Stronglyocentrotus purpuratus* and may indicate that membranes or membrane-related functions have undergone environmental selection (Oliver *et al.*, 2010). Linear relationships between such adaptations and the depth of capture in marine fish, from shallow to > 4500 m, have been interpreted as causal evidence for pressure adaptations (Cossins & Macdonald, 1986; Samerotte *et al.*, 2007). Theoretical calculations of homeoviscous adaptation in hadal organisms indicate that this is not simply temperature compensation (Somero, 1992).

Adaptations to high pressure can result in near pressure insensitivity, e.g. in malate dehydrogenases (see Somero, 1992), but it is not necessarily the case that all adaptations confer this effect. Although it is clear that some bathyal fauna can tolerate recovery from ˜2000 m and even flourish at surface pressures for up to several years (e.g. Brown & Thatje, 2011; Smith *et al.*, 2013), low-pressure intolerance has been reported for deeper bathyal and abyssal fauna (e.g. Yayanos, 1981; Treude *et al.*, 2002). This suggests that deeper living fauna may display upper bathymetric limits imposed by reduced hydrostatic pressure. These limited data appear to offer further support for a physiological bottleneck at bathyal depths.

Hyperbaric and thermal effects do appear to have made significant adaptive demands on shallow-water organisms colonising the deep sea, further supporting the influence of these factors in establishing bathymetric zonation patterns. However, it is not immediately clear how the physiological effects of hydrostatic pressure or low temperature could contribute to the unimodal pattern of diversity with depth, despite diversity typically peaking at similar depths to the proposed physiological bottleneck even though the area represented by these depths is relatively low. Is there other evidence suggesting a cause for this bathymetric diversity phenomenon?

## VI. BATHYMETRIC VARIATION IN EVOLUTION

Variation in the production of novel taxa has been identified as causative of another, somewhat analogous, evolutionary pattern in biodiversity. A meta-analysis of nearly 600 latitudinal biodiversity gradients assembled from the literature has corroborated the high generality of the latitudinal diversity decline, including for the marine environment of both hemispheres (Hillebrand, 2004*a*,*b*), a phenomenon which has prompted much discussion (e.g. see Rohde, 1992). Mid-domain effects have been shown to be inconsistent with these broad-scale patterns of species richness (Currie & Kerr, 2008). Recent assessment of the global patterns and predictors of marine biodiversity identified sea surface temperature as the only environmental predictor related to diversity across all taxa examined (Tittensor *et al.*, 2010), in agreement with the ‘out of the tropics’ dynamic (Jablonski, Roy & Valentine, 2006) and the time hypotheses (Mittelbach *et al.*, 2007). Since biodiversity patterns appear to be driven by speciation rates (Allen & Gillooly, 2006), kinetic effects of temperature on rates of genetic divergence and speciation have been proposed as the mechanism by which temperature plays a fundamental role in structuring cross-taxon marine biodiversity (Allen, Brown & Gillooly, 2002; Brown *et al.*, 2004; Allen *et al.*, 2006; Tittensor *et al.*, 2010). These are likely manifested through the effects of metabolic rate on generation time (Thomas *et al.*, 2010) and mutation rate (Gillooly *et al.*, 2001, 2005; Savage *et al.*, 2004; but see Held, 2001; Lanfear *et al.*, 2007), two fundamental variables influencing the rate of evolution (Kimura, 1983). Studies of incipient speciation and microevolution have shown faster rates of microevolution in marine foraminiferans, plants and mammals occupying low latitudes (Allen *et al.*, 2006; Wright, Keeling & Gillman, 2006; Gillman *et al.*, 2009); phylogenetic and palaeontological evidence on rates of diversification and origination also support this hypothesis (Mittelbach *et al.*, 2007). It has been argued that tropical diversification is further increased by positive feedback from sympatric speciation once standing diversity reaches a particular threshold (Briggs, 2003, 2007). Despite the complex Cenozoic history of the marine environment, tropical origination rates have left a permanent mark on the taxonomic and biogeographic structure of the modern biota (Krug, Jablonski & Valentine, 2009), including the deep sea (e.g. Macpherson *et al.*, 2010). Clearly, although it may have some bearing on latitudinal gradients that have been reported in deep-sea species diversity (Rex *et al.*, 1993), such a temperature-dependent evolutionary-rate mechanism is unlikely to explain the unimodal bathymetric pattern of diversity alone since temperature typically decreases with increasing depth. Other factors have been suggested to influence speciation rates that may be ecologically relevant in a deep-sea context (see McClain, Rex & Etter, 2009), however little consideration has been made of the potential role of hydrostatic pressure. Despite depth often being the best predictor of diversity, and apparently contributing to an evolutionary biodiversity bottleneck, it is generally believed that depth is not itself a primary driver of diversity (Levin & Dayton, 2009).

Although evolutionary rates reflect the interplay of mutation with selection and genetic drift (Kimura, 1983; Baer, Miyamoto & Denver, 2007), molecular and morphological analyses across a range of invertebrate taxa have indicated that there is greater potential for population differentiation and speciation at bathyal depths between ˜500 and ˜3300 m (France & Kocher, 1996*a*,*b*; Chase *et al.*, 1998; Etter *et al.*, 1999, 2005, 2011; Kojima *et al.*, 2001; Quattro *et al.*, 2001; Oliver *et al.*, 2010; Syme & Oakley, 2012). For example, speciation in cylindroleberidid ostracods living deeper than 1000 m is estimated to be twice as rapid as in cylindroleberidids living shallower than 1000 m (Syme & Oakley, 2012). There are persistent suggestions of cryptic speciation, which may be consistent with directional selection on ecological traits and is suggested to be non-random with regard to biome (Bickford *et al.*, 2007), in addition to reports of strong genetic variation over relatively small distances, consistently associated with differences in depth (Doyle, 1972; Siebenaller, 1978; Bucklin, Wilson & Smith, 1987; France, 1994; France & Kocher, 1996*a*, *b*; Creasey *et al.*, 1997, 2000; Chase *et al.*, 1998; Creasey & Rogers, 1999; Etter *et al.*, 1999, 2005, 2011; Morita, 1999; Kojima *et al.*, 2001; Quattro *et al.*, 2001; France & Hoover, 2002; Goffredi *et al.*, 2003; Rogers, 2003; Weinberg *et al.*, 2003; Howell *et al.*, 2004; Le Goff-Vitry, Rogers & Baglow, 2004; Held & Wägele, 2005; Zardus *et al.*, 2006; Raupach *et al.*, 2007; Brandão, Sauer & Schön, 2010; Cho & Shank, 2010; White, Stamford & Hoelzel, 2010; Boyle, 2011; Ingram, 2011; Miller *et al.*, 2011; Morrison *et al.*, 2011; Schüller, 2011; White, Fotherby & Hoelzel, 2011; Baco & Cairns, 2012; Knox *et al.*, 2012; Quattrini *et al.*, 2013). Although many shelf fauna appear to penetrate to great depth in the Antarctic (Brey *et al.*, 1996), it has been suggested recently (Rogers, 2007) that cryptic species with distinct bathymetric ranges (e.g. Held & Wägele, 2005; Raupach *et al.*, 2007; Brandão *et al.*, 2010; Schüller, 2011) may challenge the concept of extended eurybathy reported for Antarctic fauna (Brey *et al.*, 1996). Indeed, as genetic analyses proliferate cryptic speciation is increasingly reported in the Antarctic fauna (e.g. Allcock *et al.*, 1997; Rogers, Clarke & Peck, 1998; Page & Linse, 2002; Held, 2003; Raupach & Wägele, 2006; Linse *et al.*, 2007; Raupach *et al.*, 2007; Wilson *et al.*, 2007; Hunter & Halanych, 2008; Lörz *et al.*, 2009; Krabbe *et al.*, 2010) on the depressed continental shelf (average depth 450 m, extending, in places, to over 1000 m; Clarke & Johnston, 2003). It has been suggested that Antarctica may be a hotspot for this phenomenon (Grant *et al.*, 2011). By contrast, extremely low genetic diversity has been identified in abyssal organisms (Bisol, Costa & Sibuet, 1984; France & Kocher 1996*a*,*b*; Etter *et al.*, 2011). Similar trends have been reported in phenotypic variation (Etter & Rex, 1990; Rex & Etter, 1990, 1998; Rex *et al.*, 1999). There are also suggestions of low mutation rates in the deep-sea lithodid crab sub-family, Lithodinae (Hall, 2010). This pattern has prompted the proposition that the continental margins may be the primary site of adaptive radiation in the deep sea (Etter *et al.*, 2005) and the establishment of the ‘depth-differentiation hypothesis’ focusing on spatial and temporal environmental heterogeneity as the primary driver of evolution (Etter *et al.*, 2011). Links have been proposed between genetic diversity and species diversity with congruent patterns of phenotypic and genetic divergence (see Rex & Etter, 2010, and references therein). The implied elevation in speciation rate at bathyal depths between ˜500 and ˜3000 m would subsequently lead to higher diversity (Mittelbach *et al.*, 2007), consistent with the unimodal bathymetric biodiversity pattern reported for, for example, the lithodid king crabs (Zaklan, 2002; Hall & Thatje, 2009; McLaughlin *et al.*, 2010), and notothenioid (Clarke & Johnston, 1996) and macrourid fishes (Merrett & Haedrich, 1997, and references therein).

Over time a bathyal peak in evolutionary rate clearly could result in a unimodal pattern of diversity peaking at these depths, despite the relatively low area they represent. Consequently, it appears a distinct possibility that an evolutionary role for high hydrostatic pressure and low temperature may have been neglected. Indeed, reanalysis of existing diversity–depth data using quadratic depth and temperature functions may offer evidence for such a role. But how could these factors stimulate the rate of evolution at bathyal depths?

## VII. THE STRESS-INDUCED EVOLUTIONARY MECHANISM IN THE DEEP SEA

Existing evidence suggests that adaptive radiation is the predominant mode of biological diversification (see Glor, 2010, and references therein). It seems apparent from species invasions that adaptive change can occur rapidly and that severe population bottlenecks do not preclude rapid adaptation (Sax *et al.*, 2007). Links between evolutionary innovation and environmental stress have been proposed several times (see Jablonski, 2005) and molecular evidence has suggested potential mechanisms for such a stress–novelty link. Intragenomic site-specific mutation rates can vary across orders of magnitude and it has subsequently been suggested that mutation rates may be higher in sequences critical for adaptation, leading to rapid divergence even among closely related species (King & Kashi, 2009). It has been proposed that the absence of a stress response during embryogenesis and subsequent increased mutation in germ cells resulting from DNA damage may accelerate evolutionary processes (Epel, 2003). Although adaptation can arise due to a new mutation (see Rosenberg, 2001, and references therein), most adaptive alleles among identified adaptive loci seem to have been present as standing genetic variation (see Stapley *et al.*, 2010, and references therein). Bathymetric macroecological patterns may also derive from the stress–novelty link through inactivation of a canalisation system by physiological stresses during embryonic or larval development, induced by the effects of high hydrostatic pressure and low temperature around the suggested physiological bottleneck at bathyal depths (Tables 1 and 2; Fig. 2). This adaptive canalisation has been suggested to occur in extreme environments (Eshel & Matessi, 1998); the physiological effects of bathyal hydrostatic pressures and temperatures suggest that the deep sea constitutes such an environment for shallow-water species (Hall & Thatje, 2009; Thatje *et al.*, 2010; Brown & Thatje, 2011).

The ubiquitous cellular stress response affords cells a transient increase in tolerance to any form of damage-inflicting environmental challenge in larval and adult organisms, allowing time for stressor-specific adaptation to re-establish cellular homeostasis (Kültz, 2003, 2005). Such adaptive variation may be achieved by a single amino acid substitution in a protein and in response to only moderate environmental change (Somero, 2012). Exposure to high pressure has been shown to trigger an increase in the expression of stress proteins in organisms adapted to atmospheric pressure (Welch *et al.*, 1993; Takahashi *et al.*, 1997; Kaarniranta *et al.*, 1998, 2000, 2003; Elo *et al.*, 2000, 2003, 2005; Sironen *et al.*, 2002; Wemekamp-Kamphuis *et al.*, 2002), apparently as a sustained response (Cottin *et al.*, 2012). It has also been suggested that molecular chaperones such as these stress proteins, expressed when an organism is exposed to environmental extremes (Feder & Hofmann, 1999), can act as evolutionary capacitors regulating hidden variation or mutagenic activity, occasionally resulting in adaptive phenotypes (e.g. Rutherford & Lindquist, 1998; Bergman & Siegal, 2003; Madlung & Comai, 2004; Sangster, Lindquist & Queitsch, 2004; Jarosz & Lindquist, 2010; Chen *et al.*, 2012; for review see Jarosz, Taipale & Lindquist, 2010; Taipale, Jarosz & Lindquist, 2010). Although the absence of a heat shock response has been reported for some Antarctic marine invertebrates in response to temperature (Clark, Fraser & Peck, 2008*c*), this is not a universal phenomenon (Clark, Fraser & Peck, 2008*b*; Clark *et al.*, 2008*d*, 2011[Bibr b60],[Bibr b61]) and has been attributed to constitutively high levels of inducible isoforms (Place, Zippay & Hofmann, 2004; Place & Hofmann, 2005; Clark *et al.*, 2008*a*) maintaining the possibility of contribution to a stress–evolution mechanism in that habitat. Indeed, given the unusually deep Antarctic continental shelf, the stress–evolution mechanism induced by high pressure and low temperature may also contribute to the high diversity and cryptic speciation among the taxa present in the Southern Ocean relative to latitudinal trends (Brandt *et al.*, 2007; Grant *et al.*, 2011), perhaps in concert with frequent fluctuation in the extent of the grounding line of the continental ice sheet across the continental shelf during Late Cenozoic glacial periods (Clarke & Crame, 1997; Thatje *et al.*, 2005). Environmental stress may also activate or release transposable elements and it has been argued that these represent a source of significant evolutionary innovation (e.g. McClintock, 1984; McDonald, 1990, 1995; Kidwell & Lisch, 1997, 2001; Shapiro, 1999, 2005; Lisch, 2009; Casacuberta & González, 2013). Exposure of organisms to high pressure has resulted in such mobilisation of transposable elements (Aertsen & Michiels, 2005; Lin *et al.*, 2006), and alteration of methylation patterns of mobile elements has also been reported following hydrostatic pressurisation (Long *et al.*, 2006).

**Fig. 3:**
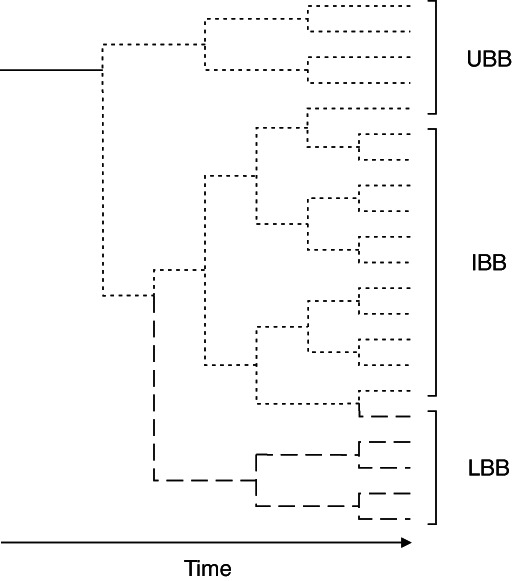
Scenario for colonisation of the deep sea following dysoxic mass extinction. An enduring upper boundary biota (UBB) species extends its distribution downslope to a taxon-specific maximum depth at the physiological bottleneck determined by interacting effects of high hydrostatic pressure and low temperature (solid line). At this limit hyperbaric and thermal stress increases mutation in germ cells, inactivates canalisation during embryonic or larval development, releases hidden genetic variation or increases mutagenic activity, or activates or releases transposable elements in larval or adult organisms, increasing genetic or phenotypic variation. Greater variation unrelated to hydrostatic pressure or temperature tolerance results in significantly increased parapatric or sympatric speciation of UBB species or inter-boundary biota (IBB) species into niches left vacant by mass extinction (dotted lines). These remain bathymetrically constrained by the combined effects of high hydrostatic pressure and low temperature. Species remain under stress promoting continuing elevated variation, and speciation rate remains increased. Variation increasing tolerance of high hydrostatic pressure or low temperature instead results in parapatric or peripatric speciation of lower boundary (LBB) species (dashed lines). Increased tolerance of high hydrostatic pressure or low temperature diminishes the stress effect and returns variation to background rate. The differential rates of speciation over time result in the unimodal pattern of biodiversity with depth. Experimental evidence indicates consistently that critical pressure and temperature conditions for shallow-water benthic invertebrate species equate to the bathyal environment, and the unimodal diversity–depth pattern typically peaks at these depths.

Mutation in germ cells, adaptive canalisation during embryonic or larval development, release of hidden genetic variation or mutagenic activity, or activation or release of transposable elements in larvae or adults, increase genetic or phenotypic variation. Elevated variation unrelated to hydrostatic pressure tolerance may promote increased parapatric or sympatric speciation into vacant niches, whilst taxa remain bathymetrically constrained (Fig. 3). Models of range restriction by gene flow along gradients, in the absence of sharp environmental boundaries, suggest increased adaptation in peripheral populations in the absence of competition, as may have been the case during colonisation of the deep sea following mass extinctions (see Carney, 2005, and references therein). By contrast, variation that results in increased tolerance of hydrostatic pressure may promote parapatric or peripatric speciation past the high hydrostatic pressure and low temperature induced bottleneck at bathyal depths, simultaneously reducing environmental stress and the subsequent evolutionary response, returning speciation to the background rate or perhaps even constraining it further. These varied forms of evolution constitute important sources for marine biodiversity (Briggs, 2006). Affected genes may represent speciation genes (see Nosil & Schluter, 2011). Such speciation would be consistent with the ecological hypothesis of speciation (Schluter, 2001). Under such circumstances elevated rates of evolution may occur at genus and species level at bathyal depths. This is consistent with elevated origination observed on the deep continental margin (Fig. 3), yielding a unimodal pattern of diversity with depth. High incidence of cryptic repeated elements in regions flanking microsatellites, which are associated with transposable elements, has been reported in examined genomes of deep-sea galatheid squat lobsters (Bailie, Fletcher & Prodöhl, 2010), hinting at recent elevated transposable element activity or mutation. Analysis of the genus *Paramunida* suggests a period of rapid diversification following origination between 17 and 21 Ma (Cabezas *et al.*, 2012), and the deep-sea galatheids of the Pacific Ocean continental slope display a unimodal pattern of diversity peaking at around 650 m (Macpherson *et al.*, 2010). Stress-protein-regulated genetic variation appears to preserve phenotypic robustness in addition to providing a broad conduit to diversification (e.g. Jarosz & Lindquist, 2010), and this mechanism may also offer an explanation for lower production of ordinal-level novel taxa in the deep sea. Although the stress effects of high pressure and low temperature could be compounded by other factors, e.g. deep-water hypoxia in oxygen minimum zones, it is unlikely that stress-induced variation alone is responsible for bathymetric macroecological patterns. Any effects on variation could be enhanced by e.g. the vicariance-mediated speciation effect proposed for transient oxygen minimum zones (White, 1987; Rogers, 2000; Levin & Sibuet, 2012), amongst other potential barriers to gene flow on continental margins (see Rex & Etter, 2010).

It is clear that understanding marine evolutionary dynamics demands increased knowledge of links between continental margin fauna (Clarke & Crame, 2010). Demonstration of intrinsic or emergent tolerance to high pressure and low temperature in taxa with well-constrained radiation and speciation from shallow water into the deep sea may ultimately help to explain the evolution of bathymetric biodiversity patterns in the deep sea. Given that we may be within a sixth mass extinction (Barnosky *et al.*, 2011) better understanding of the evolutionary impact of stress-driven adaptation is of paramount importance for assessing both the potential resilience and recovery of marine biodiversity.

## VIII. CONCLUSIONS

1. Following climate-driven dysoxic mass extinctions in the deep sea, shallow-water organisms have recolonised the deep sea and the extant deep-sea fauna appears predominantly to comprise both ancient and more recent radiations of shallow-water lineages. The physiological effects of high hydrostatic pressure and low temperature across hierarchical levels of biological organisation appear capable of limiting the distribution of such shallow-water species. Experimental assessment of hyperbaric limitation across a range of shallow-water taxa supports the proposition of a physiological bottleneck at bathyal depths, imposed by the combined effects of high hydrostatic pressure and low temperature. Organisms inhabiting the deep sea appear to be functionally adapted to the high-pressure and low-temperature conditions that prevail, suggesting that hyperbaric and thermal effects have made significant adaptive demands on shallow-water organisms colonising deeper water. Together this supports the hypothesis that a hyperbaric and thermal bottleneck at bathyal depths contributes to bathymetric zonation.
2. A unimodal pattern of diversity with depth typically peaks at similar depths to the proposed physiological bottleneck. It is recognised that speciation rates contribute to a similar latitudinal pattern in shallow-water diversity. Existing molecular and morphological evidence supports the proposition that bathyal depths are the primary site of adaptive radiation in the deep sea. We hypothesise that a peak in speciation rates at bathyal depths could establish the unimodal bathymetric biodiversity pattern over time.
3. We hypothesise that demonstrated physiological effects of high hydrostatic pressure and low temperature may promote a stress–novelty evolutionary mechanism, increasing mutagenic activity in germ cells, inactivating canalisation during embryonic or larval development, releasing hidden variation or mutagenic activity, or activating or releasing transposable elements in larvae or adults. In this scenario speciation rate is increased at bathyal depths resulting in production of novel taxa. Adaptation that increases tolerance to high hydrostatic pressure and low temperature allows colonisation of abyssal depths and reduces the stress–evolution response, consequently returning speciation in deeper taxa to the background rate. Over time this mechanism could contribute to the unimodal diversity–depth pattern.
